# Dissemination and Implementation Science for Public Health Professionals: An Overview and Call to Action

**DOI:** 10.5888/pcd15.180525

**Published:** 2018-12-20

**Authors:** Paul A. Estabrooks, Ross C. Brownson, Nicolaas P. Pronk

**Affiliations:** 1 Department of Health Promotion, College of Public Health, University of Nebraska Medical Center, Omaha, Nebraska; 2 Prevention Research Center in St. Louis, Brown School, Washington University in St. Louis, St. Louis, Missouri; 3 Department of Surgery (Division of Public Health Sciences) and Alvin J. Siteman Cancer Center, Washington University School of Medicine, Washington University in St. Louis, St. Louis, Missouri; 4 HealthPartners Institute, Bloomington, Minnesota; 5 Harvard T.H. Chan School of Public Health, Department of Social and Behavioral Sciences, Boston, Massachusetts

## A Selective Review of the Origins of Dissemination and Implementation Science

*Preventing Chronic Disease* has a mission to enhance communication between researchers, public health professionals, and policy makers to integrate research and practice experience with a goal of improved population health. As a result, those involved in dissemination and implementation (DI) science — a growing field of study that examines the process by which scientific evidence is adopted, implemented, and sustained in typical community or clinical settings — have submitted and published their rigorous and relevant work in the journal with a high degree of success. Over the previous 2 years, the journal also added a new article type — Implementation Evaluation — to facilitate submission of articles that examine the implementation of evidence-based public health interventions in community and clinical settings. In an effort to continue the focus on DI, we wrote this commentary with the following objectives: 1) to provide a brief DI description, 2) to demonstrate the shared systems–based focus of DI science and public health practice, and 3) to highlight pathways to move public health–focused DI science forward. We reflect on our own learnings and by doing so hope to motivate more public health researchers and practitioners to engage in DI research.

DI research emerged — by name — over the past 25 years (1), but its roots can be traced to a much earlier time (2–4). A review of current DI research areas likely would not have seemed out of place in the 1930s through the 1960s. Some examples include the need for clinically relevant and community-relevant research (5), engaging systems and communities as partners in the co-creation of evidence (6), and examining the characteristics of interventions to determine which are more likely to be taken to scale and sustained (7). These topics can be traced back to the origins of action research in the 1940s, the push and pull between pure and applied research in the 1960s, and the diffusion of innovations that spanned both those periods. Indeed, the works of Kurt Lewin (8), Archie Cochrane (9), and Everett Rogers (3,10) provide a strong foundation for DI science.

Kurt Lewin founded the field of action research (4,8). He and other scientists of his day struggled against a paradigm that did not consider practice professionals in the development, implementation, and interpretation of scientific studies. In a critique that sounds like it could have come from the last American Public Health Association annual meeting, Lewin criticized the lack of integration of science and practice as a lost opportunity to understand group dynamics and organizational change processes while also contributing to achieving a community benefit through research. He argued for a pragmatic epistemological approach that combined social theory, experimental or quasi-experimental methods, and practice perspectives that could be used for local decision making and contribute to generalizable knowledge. He developed numerous participatory methods that engaged organizational representatives from the settings where social solutions would be applied, members of the population intended to benefit, and social scientists to collectively conduct diagnostic, participatory, empirical, and experimental action research (8). Action research, whether described as a systems-based approach, participatory dissemination, community-based participatory research, or integrated research–practice partnerships, provides a methodological basis for much of the current DI research. It also underscores the ideal outcomes of public health–focused DI research — a balance of demonstrating local impact while concurrently contributing to generalizable knowledge on how best to move evidence into practice.

Archie Cochrane — the inspiration for the thriving Cochrane collaborative (11) and the myriad of systematic reviews developed with a goal to provide a summary of evidence that can be used for health care practice and decision making — railed against the focus on pure research over applied research during the course of his career (9,12). Indeed, this quote captures his view of the existing research paradigm in the late 1940s: “I remember being advised by the most distinguished people that the best research should be utterly useless” (9 p432). Cochrane’s approach was grounded in his experience as a prisoner of war in Germany, where he provided care for thousands of soldiers and was concerned with the likelihood that he may have inadvertently provided therapies that did more harm than good because of the lack of scientific evidence for the medical approaches of the day. As a result, he became an advocate for the use of randomized controlled trials (RCTs) for practical, applied research that could contribute to health care practice in a timely manner. By the early 1970s Cochrane was advocating for systematic reviews of literature to compile the findings of research studies and allow for guideline and policy implementation across medical disciplines (2). Cochrane reviews and other systematic review approaches (13) are used broadly in DI and to support evidence-based public health (EBPH) practice as an indicator that a given intervention is either appropriate or inappropriate for broad-scale adoption, implementation, and sustainability.

Finally, Everett Rogers could be considered the Father of DI with his seminal work published in *Diffusion of Innovations* from the first edition in 1962 through the fifth and final edition in 2003 (3). With his roots in rural sociology, Rogers introduced a theoretical approach that considered the communication of an innovation, over time and through distinct channels, across a social system. He also proposed that an innovation could be described as an idea, practice, or product that is perceived as new to a social system. Rogers’s introduction of the S-shaped curve demonstrated the relative rate of adoption across early innovators and adopters with a slower rate of spread of an innovation followed by a steep increase as the early and late majority take up the innovation, followed by a slowing of the rate of adoption when system laggards (a term Rogers referred to in personal communications as one for which he wished he had come up with a less “inherently negative” label) take up the innovation.

The characteristics of an innovation — compatibility, complexity, observability, relative advantage, and trialability (how easily an innovation can be tested) — that Rogers proposed are still foundational across the primary theoretical approaches being applied in DI research (14–16). In addition to being a foundation of DI theories, Rogers’s work is the basis of many DI process models (ie, models that provide a guiding process for moving an innovation into practice) that include stages and focus on providing knowledge about the innovation, persuasion based on the innovation characteristics, selection of an appropriate innovation, testing the innovation through implementation, and confirming whether the innovation achieved the desired results for a sustainability or discontinuation decision (3,17–19). A simplified description of Rogers’s theory is the application of this decision-making process across an S-shaped curve highlighting differences in the rate individuals or settings adopt a new innovation (ie, innovators, early adopters, early majority, late majority, and laggards), where an innovation enters a social system based on the activities of innovators and early adopters and the perceived characteristics of the innovation (ie, compatibility, complexity, cost, observability, relative advantage, and trialability). It is important to note that consideration of innovation characteristics and the applicability of the innovation–decision process occurs across and within each adopter category in a social system.

In case you think that these issues are not as relevant today as they were three-quarters of a century ago, the promulgation of evidence, the lack of relevance of evidence, and the time and capacity needed for public health professionals to adapt and implement new interventions has resulted in a considerable evidence–practice gap (20). Cochrane would be thrilled with the advances in summarizing research for implementation decisions, but we speculate that he would be disheartened to know that it takes 17 years for 14% of original research to make its way to practice (21). Furthermore, some scientists have posited that the largest return on the public and private investment for the approximately $116 billion that is spent annually on biomedical research in the United States will be from DI research focused on translating currently available research on behavioral contributors to public health — tobacco use, dietary intake, and physical activity (22,23). This underscores the need for scientific advancement in speeding the translation of public health research to EBPH practice.

## Current Dissemination and Implementation Theoretical, Process, and Outcome Models

The more recent emergence of the DI field can be traced back to the early 1990s and the energy focused on developing a myriad of DI models to address the evidence–practice gap (17,24–26). To guide public health professionals in framing their DI work, a classification system was developed that arranged models into 3 primary categories — process, explanatory, or outcome models (17). Process models are those that specify steps, stages, or phases necessary to speed the adoption, implementation, and maintenance of evidence-based interventions in clinical or community settings (19). Explanatory models are theoretical approaches to DI and specify important constructs that can predict implementation outcomes and include propositions that can be tested scientifically (3). Outcome models provide a set of potential targets for DI research and allow researchers and public health professionals to plan implementation strategies for specific outcomes and to determine the level of success of a given project or initiative (27).

Most DI researchers use a process model, though few characterize the specific steps taken at each phase of a DI project (6). However, the recently published Practical Planning for Implementation and Scale-up (PRACTIS) guide is a nice example of a process model. PRACTIS was explicitly developed to provide a step-by-step approach for researchers interested in engaging in DI work relative to physical activity promotion in clinical and community settings (19). The guide directs investigators through 4 overarching steps that include 1) identifying and characterizing the implementation setting, 2) identifying and engaging key stakeholders across multiple levels within the implementation setting, 3) characterizing barriers and facilitators to implementation, and 4) problem-solving to address potential barriers. Each step includes numerous activities to complete with the ultimate goal focusing on co-creation of implementation strategies and new evidence to support future implementation initiatives. An appealing feature of PRACTIS and other process models (28–30) is that they provide a set of algorithms and pathways based on if–then questions on potential roadblocks that may be encountered during the implementation process (19).

It’s hard not to use Rogers’s *Diffusion of Innovations* as our example of an explanatory model (3). This theory has been applied broadly, and despite the label of “diffusion,” it includes many propositions and hypotheses that can be applied to proactive adoption, implementation, and maintenance research studies; at the time of our writing this article Rogers’s work had been cited nearly 97,000 times. Diffusion of Innovations concepts have been adapted and integrated into DI-specific theories in an effort to more thoroughly operationalize theoretical constructs and expand them to define the uptake, use, and sustainability of evidence-based interventions. For example, Wandersman et al’s interactive systems framework for dissemination and implementation proposes that 3 systems interact to either facilitate or inhibit research–practice translation: a delivery system, a research synthesis and translation system, and a translational support system (16). The framework provides numerous testable hypotheses, for example, that systemic readiness for adoption of an innovation is a function of the underlying motivation for adoption based on perceptions of the innovation’s relative advantage and compatibility with systems resources, the general capacity of the system to adopt new innovations (eg, transformational leadership, organizational innovativeness), and innovation-specific capacity based on systemic implementation supports and local expertise relative to the new innovation. Having explanatory theories and applying them is a critical component to move, as Lewis and colleagues recently wrote, from simply characterizing DI to advancing the understanding of the underlying mechanisms of change (25).

DI outcomes can be generally categorized as implementation, service, or client outcomes (31). One of the earliest proposed and most cited outcome models was the Reach, Effectiveness, Adoption, Implementation, and Maintenance (RE-AIM) planning and evaluation framework published by Glasgow and colleagues in 1999 (27). RE-AIM’s goal was to provide a framework that would balance the focus on internal and external validity to improve the translation of public health interventions to practice. Researchers were encouraged to consider external validity factors associated with the population intended to benefit from the evidence-based intervention when planning and evaluating a project, including reach (penetration into the population and representativeness of those exposed to intervention efforts), effectiveness (changes in health outcomes for those exposed to the intervention), and maintenance (durability of changes in health outcomes for those exposed to the intervention). Researchers were also encouraged to consider contextual factors related to adoption at the staff and setting level (penetration into the population of potential staff and organizational delivery systems and their representativeness), implementation (cost, quality, consistency of delivery), and maintenance of implementation at the staff and organization level (durability of the quality and consistency of delivery). The RE-AIM framework has evolved over 20 years to include consideration of qualitative and quantitative data, consideration of cost across RE-AIM dimensions, and possible combinations of metrics to assess public health impact (32–37).

## The Natural Overlap of Public Health and Dissemination and Implementation Science: Systems-Based Approaches

Ultimately, public health practice is about changing systems through the use of an underlying evidence base, documenting outcomes of systems change, and capturing the underlying reasons (ie, mechanisms) of why a systems change occurred to allow for replication within and across public health settings (6). It is through this lens of systems that we consider a major goal of public health practice and DI science: to accelerate the uptake of evidence-based programs, practices, and policies in public health settings. A primary challenge to public health professionals and DI researchers alike is relevance of evidence developed through research studies to the contextual reality of practice settings (38). Few evidence-based interventions can be implemented according to the same protocol, with the same resources, and the same level of expertise when translated from a research setting to a practice, system, or policy setting (39,40). Furthermore, top–down rollouts of an evidence-based intervention where talented and effective professionals are working to achieve population-level impacts can possibly inhibit innovation and lead to poor outcomes.

Systems-based, collaborative processes for DI ideally engage practice partners that contribute across the life course of any one project and often across multiple projects (6,41,42). These processes include generation of the research question, development of implementation strategies, adaptation of evidence-based interventions, selection of the research design, implementation of the research, and interpretation of the results. This process evolved from the Cochrane era, when limited evidence from different medical fields existed and the RCT was promoted as a gold standard. Indeed, an RCT is often not feasible when applied to DI projects and could inhibit intervention adoption (43,44). One characteristic of DI work is a reliance on matching research designs to specific problems and a focus on pragmatism to answer questions that can benefit the system that is partnering on the research (45). Part of the legacy of Lewin’s work can be traced to complex systems and systems-thinking tools that are foundational areas of learning for public health professionals (46). Systems thinking tools such as multisector collaboration, iterative learning processes, and transformational leadership require the opportunity for a much broader adaptability of evidence-based interventions based on the underlying principles or processes (46). Public health professionals are often conveners and organizers of a cross section of community groups interested in improving population health (47). This convening role may include a horizontal systems approach that engages all stakeholder organizations that could be involved in the implementation of an evidence-based approach (eg, employers, faith-based organizations, community centers) as well as a vertical systems approach that acknowledges the need to engage both an administrative decision maker and staff members who would ultimately be responsible for implementation (48).

A key systems-thinking tool that is used in DI research and public health practice is an iterative learning process that includes the 1) identification of priority areas or needs within a system (similar to community needs assessments completed by public health departments), 2) matching of available evidence to the identified need (community action plans), 3) piloting or implementing strategies, 4) evaluating outcomes, and 5) deciding if a strategy should be sustained, adapted, or abandoned. Within this iterative learning process, numerous things need to be considered, and much is related to how we define evidence and evidence-based practice. Beginning with evidence-based practice, evidence is both used and produced by public health professionals (6). Kohatsu defined this EBPH approach as “the process of integrating science-based interventions with community preferences to improve the health of populations” (49 p419). The concept was also expanded to focus on evidence-based principles that can be used in the context of evidence-*informed* decision making (50–53). This concept recognizes that public health decisions are based not only on research but also on the need to consider key contextual variables (eg, political and organizational factors) (54).

Evaluation of the application of evidence-based principles and processes in the context of real-world systems is key in the iterative learning process. Often, the focus of evaluation is to provide evidence that the contribution of one or a few factors make a difference among a set of predetermined outcomes while holding all other factors constant. A systems-based approach acknowledges that in public health practice, all factors, even (or especially) those that are not being measured, are dynamic rather than static and influence the context of the evaluation. In other words, evaluations of programs, practices, and policies in the field of health and well-being are complex. The introduction of complex systems science provides an opportunity to consider an approach to evaluation that optimizes the context, does not attempt to control variables that cannot be controlled, and may be helpful in evolving the field of evaluation and pragmatic DI research to become more responsive to the needs of practitioners and decision makers (55,56).

Systems-based approaches, by nature, cannot be completed without representation from the system that is intended to change (6). This approach ensures understanding of system goals, resources, and structure and is especially critical to decision making. This approach also allows for translational solutions to align with and be responsive to the organizational context. Alignment with organizational practice priorities is paramount, and the inclusion of decision makers and implementation staff allows for both a consideration of priorities and the practicality of implementation. This need for alignment means that communications inside and outside the organization need to be kept simple and couched deeply in the work so that across all affected, people understand each other. This approach also allows for systems to set milestones and criteria necessary to determine whether a new EBPH strategy should be continued, adapted, or discontinued (6).

## A Call to Action for Public Health Practice and Dissemination and Implementation Science

Throughout this article we have highlighted the sustained relevance of the work of some of the giants in our field, provided descriptions of the importance of explanatory, process, and outcome models, and outlined the common systems-focused basis for DI science and EBPH. Here, and in the Table, we have generated some recommendations and a call to action to align with a detailed scope of EBPH (57) and suggest public health professionals should 1) make decisions based on the best available, peer-reviewed evidence, 2) use data and information systems systematically, 3) apply planning frameworks that address population health and implementation outcomes, 4) engage community (and when feasible, research) partners in decision making, 5) conduct sound evaluation across population and implementation outcomes, and 6) share what is learned.

**Table:** Recommendations for Public Health Professionals to Engage in Dissemination and Implementation Research

Recommendation	Action Steps
Leverage existing system drivers to provide opportunities to advance DI science	Study the process of adoption, implementation, and sustainability of new initiatives to integrate evidence-based principles/practice within your organization. When adaptations are made to existing evidence-based approaches, report on the reasons for adaptation and the resulting characteristics of the newly adapted strategy. Keep field notes to track the process of implementation, from the selection of an evidence-based approach to the testing of the impact of the approach, and share your results as public health DI case studies or implementation evaluations. Partner with researchers whose mission is to move science forward in a way that will concurrently fulfill public health system needs (eg, establish academic health departments).
Focus on pragmatic evaluation	Use existing measures as a cornerstone of an iterative learning approach to document the success (or not) of new evidence-based strategies. Use principles of evaluability when assessing new interventions that have not been previously evaluated (74).
Identify and use an explanatory, process, and outcome model in your work	Include, but move beyond, reporting on the effectiveness of your strategy to include a description of, if it worked, why it worked, and how you did it. Using consistent models across projects will allow for comparisons important in practice but will also provide research to move the DI field forward. Use mixed methods and present the best data available to you. Qualitative data on outcomes, mechanisms that led to the outcomes, and processes that were used for implementation can move the field forward.
Develop key competencies related to DI science	Seek out opportunities to develop capacity in the 9 key competencies for public health research and practice professionals Communicate research findings Improve practice partnerships Make research more relevant Strengthen communication skills Develop research methods and measures Consider fit between evidence and practice settings Enhance fit between evidence and practice settings Increase capacity for practical research Understand multilevel context

Building and sustaining opportunities for DI science in public health practice requires a combined emphasis on developing individual and team-based skills and capabilities as well as organizational capacity (5,58). Individual skills needed cover a range of core areas including community assessment, adaptation of evidence-based programs and policies, descriptive epidemiology, implementation and action planning, and evaluation. Team-based capabilities include skills to collaborate and the ability to bring together the necessary individual skills within work groups to optimize efficiency. To complement these individual and team-based skills, key organizational capacity includes supervisors’ expectations to use EBPH, access to information resources (eg, academic journals), and a culture and climate supportive of EBPH.

As the field of DI science continues to mature, there is increasing urgency and need for new and expanded approaches for building DI research and practice capacity (59). Because many public health researchers and practitioners lack formal training in one or more core public health disciplines, on-the-job training is urgently needed to improve DI-related skills. In recognition of this need, the important role of capacity building (eg, more training and skill development among professionals) has been noted as a “grand challenge” for public health (60). Capacity for DI research has typically been built via some combination of graduate courses, degree programs, training institutes, workshops, conferences, and online resources. Using a concept mapping process (61), we identified a set of 9 essential concept clusters (Table). To apply these competencies, several large-scale DI research training programs (eg, Dissemination and Implementation Research in Health [62]), the Implementation Research Institute [63], Mentored Training for Dissemination and Implementation Research in Cancer [64]) and smaller scale regional training programs (eg, Great Plains IDeA CTR Dissemination and Implementation Research Workshop [65], and the University of Colorado Designing for Dissemination Workshop [66]) have been conducted. Similarly, several ongoing practitioner training programs support capacity building (57,67). For example, the *Physical Activity and Public Health Course for Practitioners*, has shown positive benefits in building capacity to design, implement, and evaluate interventions (68).

Given that the field of DI research is relatively young, many gaps exist in the science (69). Some of the most critical issues for practitioners are two closely-related concepts: scalability and sustainability. Scalability involves efforts to follow a systematically timed, planned, and graded series of steps that cumulatively account for the continuously increasing reach or adoption of an intervention until a critical mass is attained the entire target population is engaged (70), or the efforts to increase the impact have been successfully tested in pilot or experimental projects to foster policy and program development on a lasting basis, thus addressing population health and inequalities (71). Sustainability has been described as the extent to which an evidence-based intervention can deliver its intended benefits over an extended period after external support from the donor agency is terminated (72) or as the long-term, ongoing support for a program in relation to an accepted value proposition that balances allocated resources against generated revenues or benefits and includes the confirmation of long-term program support through adequate proof of performance (70). A priority area for research focuses on how best to overcome barriers to scalability and sustainability that limit the benefits of evidence-based practices (73). To date, much of DI practice and research has focused on initial uptake by early adopters of one health intervention at a time. Public health professionals are in a unique position to address challenges of scalability and sustainability with a systems approach, supporting uptake and maintenance of EBPH in complex community settings that serve vulnerable populations.

In summary, a rich body of research knowledge is not moving into the hands of practitioners and policy makers as quickly and efficiently as needed. The DI approaches outlined here can begin to speed up this translation. In doing so, we will more effectively apply EBPH approaches that will use resources more efficiently, account for dollars spent, and increase impact. We encourage public health professionals — in their day-to-day work — to generate evidence that is relevant and describes how best to implement new evidence-based strategies, report on the reasons why those strategies worked, and track the effect of those strategies on implementation and population health.
